# Digital Training for Lay Health Care Workers’ Knowledge and Skills in HIV Index Case Testing: Cluster Randomized Trial

**DOI:** 10.2196/89942

**Published:** 2026-07-15

**Authors:** Tapiwa A Tembo, Nora E Rosenberg, Katie Mollan, Maria Kim, Sarah E Rutstein, Angella M Mkandawire, Mike J Chitani, Caroline Kumbuyo, Duncan Phiri, Mtisunge Mphande, Elijah Kavuta, Samuel Chilala, Jiayu Wang, Saeed Ahmed, Katherine Simon, Linda-Gail Bekker

**Affiliations:** 1 Baylor College of Medicine Children's Foundation Malawi, Off Mzimba Road, P/Bag B-397, Lilongwe, Malawi, 265 999362440; 2 Gillings School of Global Public Health, University of North Carolina at Chapel Hill, Chapel Hill, NC, United States; 3 School of Medicine, University of North Carolina at Chapel Hill, Chapel Hill, NC, United States; 4 Department of Pediatrics, Global Health Division, Baylor College of Medicine, Houston, TX, United States; 5 The Desmond Tutu HIV Centre, Institute of Infectious Disease and Molecular Medicine, Faculty of Health Sciences, University of Cape Town, Cape Town, Western Cape, South Africa

**Keywords:** index case testing, implementation fidelity, health care workers, HCWs, lay health workers, blended learning, digital training, e-learning, low- and middle-income countries, LMICs, Malawi

## Abstract

**Background:**

Task shifting in low-resource settings requires lay health care workers (HCWs) to provide a variety of health services, such as HIV index case testing, whereby sexual partners and family members of people living with HIV are offered HIV testing. For this, lay HCWs require adequate specialized training. Digital technologies hold promise for training lay HCWs in low-resource settings, but their impacts on improving knowledge, attitudes, and skills are not understood.

**Objective:**

This study evaluates the impact of digital training on lay HCWs’ knowledge, attitudes, and skills to provide HIV index case testing.

**Methods:**

We recruited lay HCWs from 34 health facilities in Malawi. We conducted a 2-arm cluster randomized controlled trial from 2022 to 2023, evaluating the impact of a digital training approach. Health facilities (clusters) were randomized 1:2 to the enhanced or standard arms. Lay HCWs in both arms received the standard in-person index case testing training. In addition, lay HCWs in the enhanced arm received tablet-guided training. Knowledge acquisition was measured using multiple-choice questionnaires administered before and after training. Attitudinal gains were assessed through a questionnaire with Likert scale responses before and after training. Between-arm mean differences were evaluated using generalized estimating equations. Skills (fidelity to index and contact testing protocols) were measured using 15-item checklists. Fidelity scores were compared between the enhanced and standard arms by estimating mean differences and 95% CIs using generalized estimating equations.

**Results:**

We enrolled 306 lay HCWs, with 125 (40.8%) in the enhanced arm and 181 (59.2%) in the standard arm. In the enhanced arm, there was 100% completion of the digital portion, 98% (123/125) completion of the face-to-face tablet-guided portion, and 81% (101/125) to 93% (116/125) completion of quality improvement sessions. Knowledge improved by 4.4% (95% CI 0.7%-8.2%) more in the enhanced arm than in the standard arm (*P*=.02). Attitudes toward digital training improved by 4.5% (95% CI 0.3%-7.0%) more in the enhanced arm than in the standard arm (*P*=.03). Lay HCWs’ fidelity to index client counseling protocols was 30.5 (95% CI 26.0%-35.0%; *P*<.001) percentage points higher in the enhanced arm than in the standard arm. Fidelity to contact client counseling protocols was 24.0 (95% CI 20.6%-27.3%; *P*<.001) percentage points higher in the enhanced arm than in the standard arm.

**Conclusions:**

Digital training improved lay HCWs’ knowledge, attitudes, and skills surrounding index case testing counseling. These findings support digital training as a useful strategy for strengthening the capacity of lay HCWs in low-resource contexts.

## Introduction

The deficit of health professionals in low- and middle-income countries (LMICs) has laid the foundation for task shifting—delegation of medical and health service responsibilities from certified professionals to lay health care workers (HCWs) [1,2]. Lay HCWs are people working at the interface between primary health care and communities to provide services [3]. Lay HCWs have contributed to improved health outcomes in maternal and child health, HIV, and noncommunicable diseases [4-6]. Adequate and regular training facilitates the acquisition of sustained knowledge, attitudes, and skills to provide quality health care services and is needed for lay HCWs to be effective [3,7,8].

Traditional synchronous centralized classroom face-to-face training has been a longstanding method of training HCWs, and it offers numerous benefits, such as face-to-face interaction and immediate feedback [9]. However, many challenges surround its provision, including a shortage of skilled trainers and infrastructure, breaks in care, poor fidelity to training content, and costs [10]. Additionally, one-time face-to-face training does not ensure retention of knowledge and skills over time [11]. Notably, failure of lay HCW programs has often been due to inadequate training and lack of regular refresher training [12,13].

Given advances in digital technology, the World Health Organization recommends digital training to expand access, and digital training may also contribute to the effective training of lay HCWs [1]. Digital training allows learning to transcend boundaries of space and time [4], limiting the time health workers spend away from work while still providing knowledge and skills [14-16]. Digital training approaches may overcome costs associated with travel, lodging, arranging centralized classroom space, and requirements for skilled trainers [7,17].

Despite the promise of digital training for lay HCWs, the impacts of digital training on reactions, knowledge, attitudes, and practices are not well understood. Although studies have evaluated the impact of digital training on knowledge, these studies were mainly descriptive, small, and not rigorously conducted [18,19]. Additionally, fewer studies have reported the impact on attitudinal gains and skills attainment in LMICs. To address these gaps, we developed and evaluated a digital training program to improve lay HCWs’ knowledge and skills in HIV index case testing [15]. This analysis is part of the PRACTICE (Package of Resources for Assisted Contact Tracing: Implementation, Costs, and Effectiveness) study, which examined the implementation and impacts of a digital training approach compared to the standard of care for Malawi’s HIV index case testing program. This analysis evaluates the impact of digital training on lay HCWs’ knowledge, attitudes, and skills.

## Methods

### Study Setting: Malawi’s Index Case Testing Program

The study was conducted in Malawi, a country in southeastern Africa with an HIV prevalence of 8.9% [20]. Nearly 60% of Malawians cannot operate a computer or internet-based mobile device [21]. Malawi is among the countries with the lowest number of clinicians per capita [22]. Hence, to address its HIV burden with limited trained human resources, Malawi has task-shifted HIV testing services to lay HCWs [23,24].

The Ministry of Health (MOH), in collaboration with United States Government partners, implemented a national lay HCW program to address the HIV epidemic. The primary responsibilities of lay HCWs include conducting HIV testing and counseling, linking persons to treatment and prevention services, tracing clients with missed clinic appointments, and collecting dried blood spot samples for viral load testing. In 2011, the role of lay HCWs expanded to implement index case testing, an evidence-based intervention where persons with HIV are counseled about recruiting their sexual partners, biological children, and other family members for HIV testing.

PRACTICE was conducted in 34 health facilities in 2 districts: Machinga and Balaka. These facilities were operated by the MOH or a faith-based organization called the Christian Association of Malawi.

The Tingathe program supported MOH and Christian Association of Malawi health facilities in the 2 districts with the provision of HIV testing services according to MOH guidelines. The Tingathe program, a PEPFAR implementing partner, is part of the Baylor College of Medicine Children’s Foundation Malawi, providing free technical assistance and support for HIV testing and treatment services [5,25]. The program employed and supervised over 95% of the HCWs providing HIV testing and care services in the study districts.

Lay HCWs at the 34 health facilities conducted HIV index case testing according to Malawi MOH guidelines [26]. Index case testing has been offered as a choice in routine HIV testing services since 2011, with concerted implementation since 2017 with United States Government implementing partners’ support [26-28]. Lay HCWs elicit contacts (sexual partners and biological children) from index clients (people living with HIV) and then provide index clients with a choice of passive referral, provider referral, contract referral, or dual referral [29].

Synchronous trainer-led face-to-face training had typically been provided for lay HCWs in Malawi’s index case testing program. However, these training modalities posed challenges, including scheduling, service disruption, high costs due to travel, lodging, and allowances, inconsistent training fidelity, and limited time for practice or feedback to reinforce skills. The PRACTICE study was designed to evaluate whether a decentralized digital training could overcome some of these challenges.

### Study Design and Population

Two of the 34 nearby small facilities were combined to form a cluster to make a total of 33 clusters. We conducted a cluster randomized controlled trial with 33 clusters (health facilities) randomly assigned in a 1:2 ratio to the standard of care plus the digital training implementation strategy (enhanced arm) or standard of care (standard arm), respectively. The digital training occurred from August to September 2022. The primary data collection period was from October 2022 to September 2023.

Research staff recruited lay HCWs between May and July 2022 who were aged 18 years or older, worked at 1 of the 34 facilities, and provided index case testing services. All lay HCWs at the facility who met the eligibility criteria were offered the opportunity to consent and enroll. Enrolled lay HCWs participated in the following activities relevant to this analysis: (1) surveys conducted before and after the training period and (2) fidelity assessments conducted after the training period. Social harms were evaluated during the study implementation.

### Implementation Strategies

#### Standard Arm

Prior to the start of the study, all lay HCWs in both arms received a 2-hour standard index case testing training, which was part of a larger in-person Tingathe training on all job responsibilities. The training included an overview of index case testing processes, referral methods, and counseling concepts. This was supplemented by routine in-person monthly facility mentorship visits conducted as part of standard implementation. The study did not alter any part of the existing standard strategies, and lay HCWs in both arms received this in October 2021.

#### Enhanced Arm

**Figure 1.**
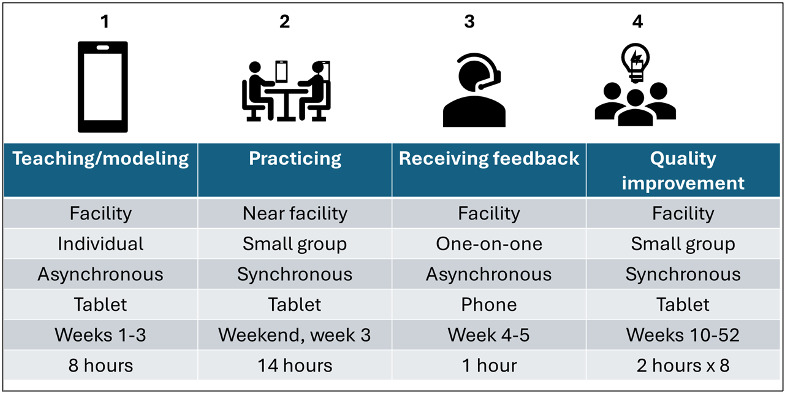
Enhanced implementation package.

The enhanced arm had 4 components: (1) teaching, (2) practice, (3) feedback, and (4) quality improvement (Figure 1) [30].

1. Teaching/modeling: The 5 self-paced digital training sessions, lasting approximately 8-hours over a 3-week period, were accessible offline and focused on teaching and modeling pedagogies. The training videos included narration of index case testing concepts and culturally appropriate dramatic scenes modeling counseling sessions of index and contact clients. The videos centered on a fictional woman newly diagnosed with HIV and her contact clients (sexual partners, children, and household members). The video production was supported by a professional videographer.
2. Practice: The 8 small group face-to-face training sessions took place at the facility or a nearby venue during the weekend for nearly 14 hours, focusing on practicing that occurred at the facility or a nearby venue.
3. Feedback: One virtual phone feedback session at the facility lasting for approximately an hour.
4. Quality improvement: Six 2-hour tablet-guided monthly quality improvement sessions at the facility that focused on the facility’s index case testing indicators to identify gaps and actionable solutions [31]. This digital training package was designed based on formative research [32], integrated social cognitive theory [33], and the theory of expertise [34]. The training was preloaded on a tablet distributed to the enhanced arm facilities and was accessible offline.

### Evaluation Framework and Outcomes

We used the Kirkpatrick training evaluation model [35] to guide this analysis. The framework has 4 levels of evaluation: reaction (level 1), which pertains to how participants perceived the training; learning (level 2), which relates to knowledge, attitudes, and skills (fidelity to index and contact testing protocols) based on the training; behavior (level 3), which measures the degree to which participants demonstrated a behavior change; and results as impact from training (level 4), which evaluates learning transfer to the clinical setting attributable to the training. We assessed the first 2 levels of the framework in this analysis: reactions and learning (Table 1). Levels 3 and 4 have been reported previously [30,36].

**Table 1.** Kirkpatrick framework training outcomes.

Outcome	Definition	Study arms	Timing	Measurement
Reaction (level 1)				
Engagement	Completion of the self-paced digital training portion	Enhanced arm	Posttraining	The study team monitored the completion of the self-directed digital training.
Satisfaction	Participants’ affective reactions to training	Enhanced arm	Posttraining	Participants completed a 9-item training evaluation mainly focusing on training quality, technical level, duration, and usefulness.
Perceived usefulness	Participants’ belief that the training improves their performance and was easy to use	Enhanced arm	Posttraining	Participants completed a 2-item training evaluation survey after each session using Likert scale response options on helpfulness and ease of use.
Attitude toward technology	Confidence in using a digital training platform	Enhanced and standard arms	Pretraining and posttraining	Attitudes toward digital training acceptability were adapted from Chao [37], with Likert scale response options.
Learning (level 2)				
Knowledge	Information on how to conduct index case testing	Enhanced and standard arms	Pretraining and posttraining	Twenty-one multiple-choice questions were developed related to the content of the digital training to measure understanding. The format was vignette-based questions developed by the study team. Twenty-five multiple-choice questions were developed related to the content of the Tingathe program training to measure understanding. The format was type A and vignette-based questions developed by the Tingathe program.
Skills	Fidelity to index client counseling protocols Fidelity to contact client counseling protocols	Enhanced and standard arms	Posttraining	Lay health care workers were observed by study staff counseling a mock index client based on a 15-item protocol. Lay health care workers were observed by study staff counseling a mock contact client based on a 15-item protocol.

Reaction was assessed through engagement, satisfaction, perceived usefulness, and attitude toward digital training. Engagement was examined through monitoring the completion of the self-paced digital training. Satisfaction included (1) perceived training quality, measured using a 5-point rating scale with response options and free comments after each training session; (2) technical level of the material covered during the training, measured using a 3-point “Just-About-Right” scale with response options; and (3) length of material covered in the training, measured using a 3-point “Just-About-Right” scale with response options (Multimedia Appendix 1). Perceived usefulness was evaluated through participants completing 2 questions as part of a feedback evaluation survey at the end of each training session on (1) how helpful they found the training session using a 5-point Likert scale with response options used to measure helpfulness and (2) did they find the tablet easy to use during the training session using a 5-point Likert-type scale used to measure difficulty. Attitude toward digital technology was measured through self-rated questions adapted from Chao [37], with Likert scale response options.

Learning was assessed through knowledge and skills acquisition after the training. Knowledge was examined using 2 multiple-choice questionnaires. Knowledge scores were calculated on a scale from 0 to 100%.

Skills acquisition was evaluated using two 15-item fidelity checklists developed by the study team, one for index client counseling and the other for contact client counseling (Table S1 in Multimedia Appendix 2). Each lay HCW participated in simulated fidelity assessments with mock index and contact clients. These sessions were standardized counseling encounters, with study staff simulating the role of an index or contact client. All lay HCWs took part in 2 audio-recorded fidelity assessments, one with an index client and the other with a contact client, after the training. Research staff audio-recorded the simulated sessions.

Two assessors blinded to the study arm then listened to each session and provided a score on each of the 15 items on the checklist: needs improvement (0), satisfactory (1), or excellent (2). Total scores (0‐30 points) were then calculated as percentages. A scoring criterion was developed for the assessors, and they were thoroughly trained to ensure consistency. The 2 assessors’ scores were averaged.

### Data Analysis

The data were exported into Stata software (version 19.0; StataCorp) for cleaning and analysis. Descriptive statistics were used to summarize baseline characteristics of the lay HCWs and study clusters. Additionally, descriptive analyses were performed to assess engagement, satisfaction, and perceived usefulness, as these were only ascertained in the enhanced arm.

Analyses of pretraining and posttraining knowledge and attitude questions included participants who responded to the same question at both time points. The scores were evaluated using the difference (enhanced−standard) in differences (Post-Pre within person) approach to adjust for small baseline differences between arms. The units of analysis for knowledge and attitude were the mean changes in per-item scores before training and post training. Between-arm (enhanced vs standard arm) mean differences were evaluated using generalized estimating equations with an identity link and normal distribution to account for potential correlation between observations at the cluster level.

Skill acquisition posttraining scores were used to assess agreement and/or reliability. Cohen κ with 95% CIs was calculated to evaluate scoring consistency (reliability) between the 2 assessors per checklist item. Landis and Koch [38] threshold values were used to interpret Cohen κ results. The κ values ranged from 0 (absence of agreement) to 1 (perfect agreement). Bland and Altman method [39] was used to evaluate the reliability of assessors’ scoring. Two-sample Welch *t* tests were used to compare the mean fidelity scores per item between the enhanced and standard arms. Fidelity scores with 95% CIs were compared between enhanced and standard arms using generalized estimating equations with an identity link and normal distribution to account for potential correlation between observations at the cluster level. Fidelity scores for index and contact clients were analyzed separately.

### Ethical Considerations

The study was reviewed and approved by the National Health Sciences Research Committee in Malawi (20/06/2566), the University of North Carolina at Chapel Hill Institutional Review Board (20-1810), and the Baylor College of Medicine Institutional Review Board (H-48800). Written informed consent was obtained from all study participants (Checklist 1). The trial was registered at ClinicalTrials.gov (NCT05343390). Participants received US $10 per study activity including: completing surveys, and three times for digital training activities.

## Results

### Population Characteristics

**Figure 2.**
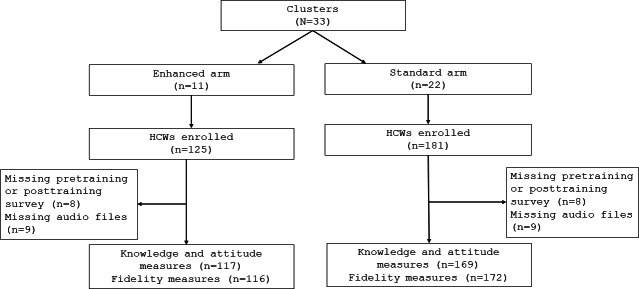
Flowchart of study participants. HCWs: health care workers.

The study was conducted in 13 clusters in Balaka and 20 clusters in Machinga. There were 33 clusters, with 11 in the enhanced arm and 22 in the standard arm (Figure 2). The majority of clusters, 26 (79%), were in rural settings. Most clusters, 27 (82%), were health centers, and a few were secondary-level hospitals or small dispensaries. The median staffing per facility was 7 (IQR 6‐9).

All the lay HCWs we approached at the facilities consented to join the study. They were all lay HCWs. We enrolled 306 lay HCWs: 125 (40.8%) in the enhanced arm and 181 (59.2%) in the standard arm. One lay HCW from the standard arm did not complete the baseline survey, and data for 305 lay HCWs are presented. The median age of the lay HCWs was 34 (IQR 27‐39) years, and the majority, 173 (57%), completed secondary education. The median time in the lay HCW role was 3 (IQR 1‐5) years. Most lay HCWs, 242 (80%), owned their own smartphone, and half had received prior tablet-based training (Table 2).

**Table 2.** Health care worker characteristics by study arm.

Characteristic	Enhanced arm (n=125), n (%)	Standard arm (n=180)^a^, n (%)
Age (y)		
21‐30	48 (38)	80 (44)
31‐40	59 (47)	54 (30)
41+	18 (14)	46 (26)
Sex		
Male	66 (52)	93 (51)
Female	59 (47)	87 (48)
Cadre		
CHW^b^	37 (30)	55 (31)
CHW/counselor	63 (50)	84 (47)
HIV diagnostic assistant	9 (7)	13 (7)
Tingathe site supervisor	12 (10)	22 (12)
Other health worker	4 (4)	6 (3)
Education		
Some secondary education	16 (13)	27 (15)
Completed secondary education	73 (58)	102 (56)
Tertiary education	36 (29)	51 (28)
Work experience (y)		
<1	24 (19)	32 (18)
1‐3	45 (36)	62 (34)
4‐6	40 (32)	65 (36)
7+	16 (13)	21 (12)
Own a smartphone		
No	19 (15)	33 (18)
Yes, mine	102 (82)	142 (79)
Yes, shared	4 (3)	5 (3)
Trained using a mobile device before		
No	64 (51)	91 (51)
Yes	61 (48)	89 (49)
Prior use of a tablet for work		
No	84 (67)	126 (70)
Yes	41 (33)	54 (30)
Prior index case testing training^c^		
No	29 (23)	42 (23)
Yes	95 (76)	137 (76)

a One lay health care worker left before completing the baseline survey.

b CHW: community health worker.

c Two lay health care workers chose not to respond.

### Summary of Key Findings

Knowledge about index testing and attitudes toward digital training improved more in the enhanced arm than in the standard arm. At endline, fidelity measures were higher in the enhanced arm than in the standard arm. We detail these findings below.

### Engagement

In the enhanced arm, 100% of the lay HCWs completed 8 hours of the self-paced digital training portion within the allotted 3 weeks. Uptake of the small group face-to-face training sessions was 98%, and the virtual phone feedback session was 100%. Quality improvement sessions ranged from 81% to 93%.

### Experience of Digital Training

Most participants expressed satisfaction with self-paced training sessions (107/125, 85%) and small-group face-to-face training sessions (112/125, 90%). Additionally, the majority of participants (108/125, 86%) agreed that the digital training and face-to-face training content technical levels were just right and provided content relevant to their practice. Furthermore, 86% (108/125) of the participants perceived the length of the course to be adequate.

The results of the training evaluation after each session in the enhanced arm, on a scale of 1 to 5, with 5 being the highest, showed that overall HCWs found the training very helpful, with a mean of 4.3 (SD 0.6). They also found it very easy to use, with a mean of 4.8 (SD 0.6).

### Attitudes Toward Any Digital Training

There were 94% (286/305) lay HCWs who completed the surveys pretraining and posttraining. On average, digital technology acceptability scores increased by 1.9%, from 67.0% pretraining to 68.9% posttraining in the enhanced arm, while in the standard arm, there was a 2.6% reduction in acceptability scores, from 67.9% pretraining to 65.2% posttraining. The between-arm difference in pre-to-post differences comparing the enhanced vs standard arms was 4.5% (95% CI 0.3%, 7.0%; *P*=.03).

### Knowledge

There were 94% (286/305) lay HCWs who completed the questions pretraining and posttraining. Knowledge improved from 63.3% pretraining to 70.4% posttraining in the enhanced arm. It was relatively consistent in the standard arm: 63.4% to 66.2%. The between-arm difference was 4.4% (95% CI 0.7%-8.2%) percentage points higher in the enhanced arm (*P*=.02). Correct answers for the Tingathe program’s index case testing examination increased from 68.2% pretraining to 72.8% in posttraining in the enhanced arm compared to no improvement in the standard arm (68.9% pretraining and 69.4% posttraining; Table 3). The between-arm difference in scores was 4.0% (95% CI 1.4%-6.7%; *P*=.002).

**Table 3.** Comparison of health care worker knowledge percentage scores and attitude scores between the enhanced and standard arms.

Item	Enhanced arm (n=117)			Standard arm (n=169)			Between-arm MD^a^	
	Pretraining	Posttraining	MD (95% CI)	Pretraining	Posttraining	MD (95% CI)	MD (95% CI)	*P* value
Knowledge: 21 multiple-choice questions developed by the study team	63.4	70.4	7.0 (4.5 to 9.5)	63.4	66.2	2.6 (0.7 to 4.4)	4.4 (0.7 to 8.2)	.02
Knowledge: 25 multiple-choice questions developed by the Tingathe program	68.2	72.8	4.6 (2.7 to 6.5)	68.9	69.4	0.6 (−0.9 to 2.0)	4.0 (1.4 to 6.7)	.002
Digital technology acceptability	67.0	68.9	1.9 (−0.5 to 4.3)	67.9	65.2	−2.6 (−5.0 to −0.3)	4.5 (0.3 to 7.0)	.03

a MD: mean difference.

### Skill Acquisition

For the index client-HCW encounters, interassessor reliability with Cohen κ ranged from 0.56 to 0.95. For the contact client-HCW interassessor reliability with Cohen κ ranged from 0.69 to 0.97 (Table S1 in Multimedia Appendix 2). The Bland-Altman plots demonstrated good agreement between the assessors (Figure S1A,B in Multimedia Appendix 3).

There were better scores across each of the 15 items in the enhanced arm compared to the standard arm for the index fidelity checklist (*P*<.001). Similarly, there were better scores across 13 of the 15 items in the enhanced arm compared to the standard arm on the contact fidelity checklist (Table S1 in Multimedia Appendix 2).

**Figure 3.**
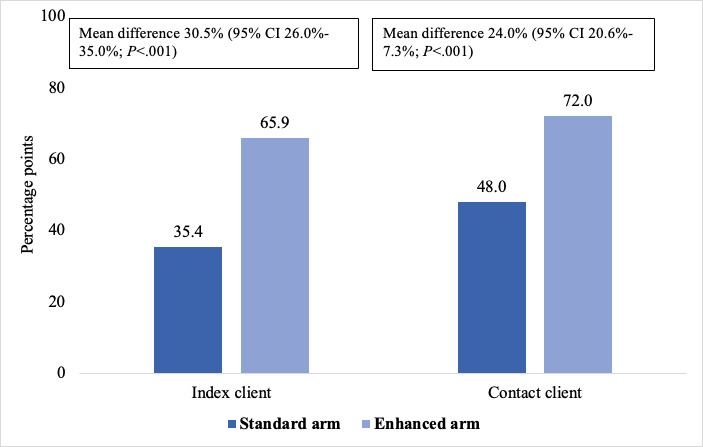
Fidelity scores between arms.

The mean fidelity score for index client-HCW encounters was higher in the enhanced arm (65.9%, 95% CI 62.1%-69.8%), compared to the standard arm (35.4%, 95% CI 33.1%-37.7%). Fidelity was 30.5 percentage points higher in the enhanced arm (mean difference 30.5%, 95% CI 26.0%-35.0%; *P*<.001). The mean fidelity score for contact client-HCW encounters was 72.0% (95% CI 69.4%-74.5%) in the enhanced arm and 48.0% (95% CI 45.7%-50.1%) in the standard arm. Fidelity was 24.0 percentage points higher in the enhanced arm (mean difference 24.0%, 95% CI 20.6%-27.3%; *P*<.001; Figure 3).

## Discussion

We developed and rolled out a digital training for lay HCWs in Malawi. Lay HCWs completed nearly the entire training; most were satisfied with it and found it easy to use. This training modestly improved knowledge (<5%) of index case testing concepts and attitudes toward digital training. Further, the training substantially improved (>20%) lay HCWs’ skills to conduct index case testing counseling.

The PRACTICE study is among the first randomized trials assessing the impact of digital training using an experimental design in a low-resource setting. Notably, previous studies were small, descriptive, and nonexperimental. A scoping review that focused on digital training for rural professional cadres [18] identified few experimental studies (n=5) and focused mainly on knowledge acquisition. Similarly, a systematic review that evaluated digital learning for medical education in LMICs [40] found only 4 randomized controlled trials. Most prior work focused on the impact of digital training on knowledge. An increase in knowledge after digital training has been found in other LMICs [4,41] and high-income countries [10,42]. Our digital training also modestly improved knowledge of index case testing protocols and principles.

This study is among the first to provide evidence for enhanced skills. Our results are consistent with a similar study from Bangladesh reporting that digital training improved HCWs’ counseling skills in the context of family planning and maternal and child health [43]. The substantial skill enhancement, coupled with only a modest increase in knowledge, suggests that knowledge was not the only ingredient needed to uplift HCWs’ skills. Although our enhanced training included some didactic knowledge, it focused on modeling, practicing, and offering feedback on counseling skills. These pedagogical elements likely drove the improvements in skills. Similarly, acceptance of technology may have been a necessary ingredient for completing training, but it was not the main driver of skill improvement. In addition, we also observed an impact on behavioral and clinical outcomes in the PRACTICE study [30,36]. Perhaps the improvement in skills is what led to improvements in behavioral and clinical outcomes.

Overall, the lay HCWs reported a high level (>85%) of engagement and satisfaction. All participants completed the digital component. Other reports from Kenya [44] and Rwanda [6] similarly found digital training acceptable and feasible due to flexibility and content relevance. This was a clear advantage over centralized training, where lay HCWs would need to schedule time off work and potentially travel to an off-site venue. The inclusive methods used to develop the training and customized attention to culture and local context may have enhanced the levels of engagement and satisfaction [45-47].

HCWs in the enhanced arm had improved acceptability toward digital training modalities compared to those in the standard arm. Our findings are consistent with other research in low-resource settings, which shows that digital training interventions to improve lay HCWs’ quality of health services can be acceptable [7,45]. Additionally, positive attitudinal gains after digital training have been reported in other studies in Malawi [48] and other LMICs [49], demonstrating that technology-based training for lay HCWs in LMICs can improve confidence and attitudes to use the technology.

We designed our training with feasibility considerations in mind. First, our choice of an *offline* platform took into consideration the limited network infrastructure in this setting. Second, we oriented the facility supervisors on how to support training for their sites. Third, we used a training platform that was easy to navigate. Finally, the allocated time to complete the training was appropriate.

One limitation of the digital training acceptability and satisfaction measures is social desirability. HCWs may have sought to please trainers who were requesting feedback, thereby providing more positive responses than they might otherwise. To minimize this, lay HCWs were encouraged to respond honestly and to provide their feedback privately.

It is not known whether the results of this study would generalize to government-employed lay HCWs, who provide services in facilities that lack the support of implementing partners. Government- and Tingathe-supported lay HCWs may differ in their scope of work, supervision, management, funding source, and compensation. Given that digital training has the potential to meet the training needs of lay HCWs with minimal disruption compared to centralized training, it is important to understand the uptake of digital training strategies in other contexts as the next step.

Contamination was a small potential risk in this study. In this study, contamination referred to HCWs in the standard arm having access to digital training. Notably, the lay HCWs were recruited from real-world settings; therefore, it was not possible to eliminate contamination. We minimized this by controlling access to the training tablets. We also tracked all staff transfers from one facility to another, and this was minimal (<1% of lay HCWs).

Digital training can be an acceptable and practical tool to enhance the competence of lay HCWs to conduct index case testing counseling in a resource-constrained setting. The training strengthened HCWs’ reaction and confidence in digital training. It led to improvement in knowledge and counseling skills, which may strengthen the provision of quality health care services. In light of reductions in development assistance for health, there is an increasing need for decentralized, cost-effective, minimal-interruption training for HCWs in LMICs, and our digital training package shows promise in supporting this need. Digital training approaches can address training needs for lay health workers in many low-income settings.

## Supplementary material

10.2196/89942 Multimedia Appendix 1 Index case testing training evaluation form.

10.2196/89942 Multimedia Appendix 2 Mean scores and Cohen κ values per item on the simulated fidelity checklists.

10.2196/89942 Multimedia Appendix 3 Bland and Altman plot of (A) index and (B) contact clients–health care worker simulated fidelity score.

10.2196/89942 Checklist 1 CONSORT-EHEALTH checklist (V 1.6.1).

## Abbreviations

CONSORT-EHEALTH: Consolidated Standards of Reporting Trials of Electronic and Mobile Health Applications and Online Telehealth
HCW: health care worker
LMICs: low- and middle-income countries
MOH: Ministry of Health
PRACTICE: Package of Resources for Assisted Contact Tracing: Implementation, Costs, and Effectiveness
