# Supplementary material for: Digital Training for Lay Health Care Workers’ Knowledge and Skills in HIV Index Case Testing: Cluster Randomized Trial
Source: JMIR Med Educ. 2026 Jul 15;12:e89942. doi: 10.2196/89942 (PMC13372267; doi:10.2196/89942)
Supplement: Multimedia Appendix 1 [file mededu-v12-e89942-s001.docx]

**Appendix 3: Index case testing training evaluation form**

**Instructions to Participant:**

- Thank you for participating in this ICT Training. Your feedback is very important to help improve the training materials and approach.

1. Please complete the following table by ticking the column of your choice for each item:

| Please rate the quality of the following: | Poor | Fair | Good | Very Good | Excellent |
| --- | --- | --- | --- | --- | --- |
| 1. Overall Individual sessions (conducted at your facility on a tablet during a weekday) |  |  |  |  |  |
| 1. Session 1: An Introduction to ICT approaches |  |  |  |  |  |
| 1. Session 2: Foundations for providing high-quality ICT services |  |  |  |  |  |
| 1. Session 3: Working with Index clients |  |  |  |  |  |
| 1. Session 4: Working with Contacts |  |  |  |  |  |
| 1. Session 5: Continuous Quality Improvement |  |  |  |  |  |
| 1. Appendices: Videos of a counselor with Barbara, Frank, and Moses, and their network |  |  |  |  |  |
| 1. Overall Face-to-face sessions (conducted over the weekend with facilitators in small groups) |  |  |  |  |  |
| 1. Session facilitation |  |  |  |  |  |
| 1. Practice counseling in pairs |  |  |  |  |  |
| 1. Discussions after practice sessions |  |  |  |  |  |
| 1. Practice quality improvement in groups |  |  |  |  |  |
| 1. Individual feedback you received from facilitators at the face-to-face sessions |  |  |  |  |  |
| 1. Index client checklist |  |  |  |  |  |
| 1. Contact client checklist |  |  |  |  |  |

2. The technical level of the material covered in the Individual training was: (circle one)

_(0)_ Too basic  _(1)_ Just right  _(2)_ Too difficult/technical

3. The technical level of the material covered in the face to face training was: (circle one)

_(0)_ Too basic  _(1)_ Just right  _(2)_ Too difficult/technical

4. The material covered in the individual training was: (circle one)

_(0)_ Too long  _(1)_ Just right  _(2)_ Too short

5. The material covered in the face to face training was: (circle one)

_(0)_ Too long  _(1)_ Just right  _(2)_ Too short

6. Please list up to 3 lessons learned during this training that you will take back to the health facility where you work.

7. What part of the training was the *most* useful for your work?

8. What part of the training was the *least* useful for your work?

9. Other Comments:

**Thank you for your feedback!**
