# Supplementary material for: Digital Training for Lay Health Care Workers’ Knowledge and Skills in HIV Index Case Testing: Cluster Randomized Trial
Source: JMIR Med Educ. 2026 Jul 15;12:e89942. doi: 10.2196/89942 (PMC13372267; doi:10.2196/89942)
Supplement: Multimedia Appendix 2 [file mededu-v12-e89942-s002.docx]

**Appendix 1: Supplementary Tables**

***Supplementary* Table 1: Mean scores and Cohen’s kappa values per item on the simulated fidelity checklists**

| **Item** | **Enhanced arm**  **Mean score (n=116)** | **Standard arm**  **Mean score (n=172)** | ***P* value** | **Kappa-value**  **(95% CI)** | **Agreement** |
| --- | --- | --- | --- | --- | --- |
| **Index client checklist** | | | | | |
| Introduction | 1.29 | 0.63 | <.001 | 0.90 (0.85, 0.94) | 98.1% |
| Confidentiality and consent | 1.63 | 0.47 | <.001 | 0.91 (0.86, 0.96) | 95.8% |
| Elicit children/guardians | 1.38 | 0.90 | <.001 | 0.80 (0.73, 0.86) | 96.5% |
| Elicit sexual partners | 1.15 | 1.04 | <.001 | 0.74 (0.61, 0.86) | 98.5% |
| Elicit household members | 1.38 | 0.26 | <.001 | 0.91 (0.88, 0.94) | 97.2% |
| Discuss referral methods | 0.64 | 0.24 | <.001 | 0.91 (0.87, 0.95) | 98.5% |
| Discuss pros/cons of each method | 1.31 | 0.67 | <.001 | 0.95 (0.91, 0.97) | 98.8% |
| Assess safety/provide IPV referrals | 0.77 | 0.22 | <.001 | 0.81 (0.72, 0.88) | 96.4% |
| Select testing/tracing method | 1.47 | 0.82 | <.001 | 0.69 (0.61, 0.75) | 92.2% |
| Support method selected | 0.94 | 0.31 | <.001 | 0.88 (0.84, 0.92) | 97.2% |
| Finalize session | 0.94 | 0.30 | <.001 | 0.81 (0.74, 0.87) | 97.1% |
| Client-centered, non-coercive | 1.56 | 0.81 | <.001 | 0.80 (0.73, 0.86) | 95.7% |
| Non-judgmental | 1.95 | 1.58 | <.001 | 0.56 (0.45, 0.68) | 94.7% |
| Culturally appropriate | 1.94 | 1.80 | <.001 | 0.56 (non-convergence) | 97.1% |
| Natural order and flow | 1.45 | 0.59 | <.001 | 0.84 (0.79, 0.88) | 95.8% |
| **Contact client checklist** | | | | | |
| Introduction | 1.83 | 1.81 | .71 | 0.81 (0.70, 0.90) | 98.4% |
| Confidentiality and consent | 1.41 | 0.32 | <.001 | 0.97 (0.95, 0.99) | 99.0% |
| Discuss previous test | 1.76 | 1.04 | <.001 | 0.97 (0.94, 0.99) | 98.9% |
| Assess engagement in care | 0.98 | 0.07 | <.001 | 0.96 (0.92, 0.99) | 98.8% |
| Discuss HIV testing | 1.11 | 0.84 | <.001 | 0.97 (0.95, 1.00) | 99.6% |
| Discuss linkage to treatment/prevention | 0.54 | 0.26 | <.001 | 0.91 (0.86, 0.95) | 98.9% |
| Conduct HIV test | 1.97 | 1.86 | .01 | 0.90 (0.78, 0.97) | 99.3% |
| Provide treatment/prevention | 1.69 | 1.17 | <.001 | 0.96 (0.92, 0.98) | 98.2% |
| Conduct index case testing | 0.83 | 0.28 | <.001 | 0.90 (0.85, 0.95) | 98.4% |
| Finalize session | 0.85 | 0.36 | <.001 | 0.81 (0.74, 0.87) | 97.4% |
| Voluntary, non-coercive | 1.59 | 0.88 | <.001 | 0.79 (0.70, 0.86) | 96.1% |
| Client-centered | 1.53 | 0.88 | <.001 | 0.69 (0.60, 0.79) | 94.8% |
| Non-judgmental | 2.00 | 1.92 | .001 | 0.94 (0.80, 1.00) | 99.8% |
| Culturally appropriate | 1.98 | 1.96 | 0.25 | 0.82 (0.33, 1.00) | 99.6% |
| Natural order and flow | 1.51 | 0.74 | <.001 | 0.88 (0.83, 0.93) | 97.7% |
