# Supplementary material for: Digital Training for Lay Health Care Workers’ Knowledge and Skills in HIV Index Case Testing: Cluster Randomized Trial
Source: JMIR Med Educ. 2026 Jul 15;12:e89942. doi: 10.2196/89942 (PMC13372267; doi:10.2196/89942)
Supplement: Multimedia Appendix 3 [file mededu-v12-e89942-s003.docx]

**Appendix 2: Supplementary Figures**

**Supplementary Figure 1a. Bland and Altman plot of index clients-HCW simulated fidelity score**

**Supplementary Figure 1b. Bland and Altman plot of contact clients-HCW simulated fidelity score**
